# Respiratory syncytial virus exhibits differential tropism for distinct human placental cell types with Hofbauer cells acting as a permissive reservoir for infection

**DOI:** 10.1371/journal.pone.0225767

**Published:** 2019-12-02

**Authors:** Vladimir Bokun, John J. Moore, Robert Moore, Carrie C. Smallcombe, Terri J. Harford, Fariba Rezaee, Frank Esper, Giovanni Piedimonte

**Affiliations:** 1 Center for Pediatric Research, Department of Inflammation and Immunity, Lerner Research Institute, Cleveland Clinic, Cleveland, Ohio, United States of America; 2 Department of Pediatrics, MetroHealth Medical Center, Cleveland, Ohio, United States of America; 3 Center for Pediatric Infectious Diseases, Cleveland Clinic Children’s, Cleveland, Ohio, United States of America; BC Children's Hospital, CANADA

## Abstract

**Background:**

Respiratory syncytial virus (RSV) is capable of transient viremia and extrapulmonary dissemination. Recently, this virus has been identified in fetal cord blood, suggesting the possibility of *in utero* acquisition in humans. Here, we assess permissivity and kinetics of RSV replication in primary human placental cells, examine their potential to transfer this infection to neighboring cells, and measure the inflammatory response evoked by the virus.

**Methods and findings:**

Human placental villus tissue was collected immediately upon delivery and processed for isolation of placental cytotrophoblast, fibroblast, and macrophage (Hofbauer) cells. Isolated cells were infected with a recombinant RSV-A2 strain (rrRSV) expressing red fluorescent protein (RFP) and analyzed by fluorescence microscopy, Western blot, and quantitative PCR (qPCR). Based on RFP expression, rrRSV exhibited differential tropism for the three major placental cell types. Placental fibroblasts and Hofbauer cells were permissive and supported productive rrRSV replication. While infected cytotrophoblast cells expressed viral glycoprotein (G protein), only limited RSV replication was detected. Importantly, qPCR and fluorescence-focused unit assay revealed that the viral progeny remains trapped within infected Hofbauer cells for up to 30 days, with no release into surrounding media. Yet, Hofbauer cells passed the infection onto overlaid naïve 16HBE cells, suggesting contact-dependent *trans*-infection. Lastly, a significant increase in proinflammatory cytokines, particularly IL-6, TNF-alpha, and IFN-gamma was measured in the supernatant of infected Hofbauer cells by multiplex cytokine assay and conventional ELISA.

**Conclusions:**

This study demonstrates that RSV can replicate in human placenta, exhibits differential tropism for distinct placental cell types, can be stored and transferred to neighboring cells by Hofbauer cells, and elicits an inflammatory response. It also supports the hypothesis that this respiratory virus can be vertically transferred to the fetus and potentially affect its development and the outcome of pregnancies.

## Introduction

There is growing evidence for extrapulmonary complications of respiratory syncytial virus (RSV) infection, including *in utero* acquisition by the fetus during pregnancy. This hypothesis first emerged from the detection of RSV antigens and genome in the peripheral circulation and extrapulmonary tissues of infected human subjects [1–5]. Later, full RSV genome was sequenced in lung tissues of 40% of the offspring born to pregnant rats infected with RSV at mid-gestation, and the virus was also found to still be present in 25% of adult rats exposed only *in utero* [6]. RSV infection of fetal lungs upregulated nerve growth factor (NGF) expression, causing post-natal airway hyperreactivity [6], and induced selective immune tolerance to postnatal reinfection with the same virus [7]. More recently, RSV genome has been amplified from human cord blood mononuclear cells, as well as from a newborn with congenital RSV infection born to a mother who contracted the virus in the third trimester of pregnancy [8, 9].

To date, the mechanisms by which respiratory viruses like RSV can spread to the fetus remain unclear. The placenta serves as both a physical and immunological barrier that effectively blocks most infectious agents from entering the fetal circulation and amniotic fluid. However, some viral pathogens exhibit tropism for distinct placental cell types and can gain access to fetal tissues. For instance, human cytomegalovirus (CMV) is capable of crossing the syncytiotrophoblast by transcytosis of immune complexes, and replicates in the underlying cytotrophoblast before spreading to the fetus [10]. Alternatively, coxsackievirus infects trophoblast cells in a lipid raft-dependent fashion [11], whereas hepatitis B virus (HBV) invades cells within the placenta as well as the decidua, including trophoblast, macrophages (Hofbauer cells), and capillary endothelium [12, 13].

After the recent discovery of congenital brain abnormalities in children infected with Zika virus (ZIKV), renewed effort has been placed on understanding the mechanisms of transplacental infections. ZIKV exhibits tropism for Hofbauer cells and placental fibroblasts, and to a much lesser extent for cytotrophoblast cells [14]. Importantly, Hofbauer cells serve as a permissive reservoir for its replication [15, 16], and because of the close proximity to umbilical cord blood vessels, these cells may also serve as a vehicle of viral dissemination into the fetal circulation [16]. Additionally, Hofbauer cells are characterized by migratory behavior within the villous stroma and make direct contact with other macrophages and stromal cells, both of which may be implicated in the transmission of ZIKV to the fetus [17]. Thus, it is now widely believed that Hofbauer cells are of central importance to the acquisition of congenital viral infections [18].

In this study, we sought to: 1) characterize RSV tropism for the three major cell types present in placental villus tissue: cytotrophoblast cells, stroma fibroblasts, and Hofbauer cells; 2) explore the potential role and the mechanisms exploited by Hofbauer cells in transmitting RSV to the fetus following maternal viremia; and 3) analyze the release of soluble proinflammatory cytokines from RSV-infected Hofbauer cells.

## Materials and methods

### Collection of placental tissue

Placental villous tissue was collected immediately upon uncomplicated, full-term (37–42 weeks’ gestation), elective C-section deliveries at MetroHealth Hospital in Cleveland, Ohio. Exclusion criteria were: mother’s age less than 18 years; clinical evidence of chorioamnionitis or intrauterine infection; history of diabetes or gestational diabetes; newborn with fetal anomalies; fetal or acute neonatal death in the delivery room; significant separation of the amnion from the choriodecidua at delivery; meconium stained amniotic fluid; multiple fetal pregnancy. Processing of tissues for the isolation of cells started within 15 min of delivery. This study was approved by the Cleveland Clinic and MetroHealth Institutional Review Boards (IRB# 16–1311 and 16–00335, respectively). We worked with normally discarded placentas with intact fetal membranes, and following inclusion in the study no protected health information, identifiers, or clinical data were collected. A waiver of consent was approved by the IRB since the placentas were collected anonymously.

### Isolation of primary placental cells

Isolation of cytotrophoblast, fibroblasts, and Hofbauer cells was performed as described previously [19] with minor modifications. This protocol yields 98–99% pure cell populations. Hofbauer cell purity was confirmed by CD163 and CD45 staining, whereas fibroblasts and cytotrophoblast purity was confirmed by visualization.

- Cytotrophoblast cells: Placental cotyledons were washed with PBS, dissected from the decidual layer, minced, and sequentially digested with 0.25% trypsin (Gibco, Gaithersburg, MD) and 0.2 mg/ml DNase I (Sigma-Aldrich, St. Louis, MO). Cells obtained from the second and third digestion were filtered through sterile gauze and a 100-μm filter, then centrifuged through a preformed Percoll gradient to remove cell debris and contaminating cells. After being washed with media to remove the Percoll, the cell pellets were re-suspended in isolation buffer (PBS with 2 mM EDTA and 0.1% BSA) and immunopurified using anti-CD9 (R&D Systems, Minneapolis, MN) and anti-CD45 (Invitrogen, Carlsbad, CA) mouse monoclonal antibodies to tag contaminating cell types. After 15 min incubation, goat anti-mouse conjugated magnetic Dynabeads (Invitrogen) were added to deplete the previously tagged cells using a magnet.
- Fibroblast cells: Placental fibroblasts were removed from Dynabeads incubated with CD9 and CD45 antibodies, plated in DMEM/F12 with 10% FBS and 1% Pen/Strep, and allowed to proliferate. Cells were then detached and exposed to a magnetic field to remove remaining bead-bound cells before re-seeding.
- Hofbauer cells: Tissue fragments remaining after trypsin digestions were washed with PBS and digested further with collagenase A (1 mg/ml) and DNase I (0.2 mg/ml). After final dissociation by gentle trituration, the resulting supernatant was filtered through sterile gauze and a 100-μm filter. After centrifugation at 1250xG for 15 min, the cells were separated using Percoll or Ficoll gradients. The resulting cell suspension was immunopurified by direct negative selection using goat anti-mouse Dynabeads pre-conjugated with anti-EGFR (Santa Cruz Biotechnology, Dallas, TX) and anti-CD10 (Invitrogen) mouse monoclonal antibodies to deplete cytotrophoblast and fibroblast cells. Finally, Hofbauer cells were plated in RPMI-1690 medium with 5% FBS for 1 hour, after which floating cells were washed off and fresh DMEM/F12 medium supplemented with ITS + Premix and 1% Pen/Strep was added.

### Culture of placental cells

Cytotrophoblast cells were cultured for up to 3 days in IMDM media supplemented with 10% FBS and 1% Pen/Strep. Hofbauer cells were grown for up to 6 days in DMEM/F12 media supplemented with ITS + premix and 1% Pen/Strep. Fibroblasts were cultured in DMEM/F12 medium with 10% FBS and 1% Pen/Strep. All cells were cultured in 6-well plates at 37°C in 5% CO_2_. Cytotrophoblast and Hofbauer cells were used at passage 0, whereas fibroblasts were passaged up to 3 times.

### Virus preparation and infection

The original stock of RSV-A2 strain expressing the red fluorescent protein (RFP) upon replication (rrRSV) was kindly provided by Dr. Mark Peeples (Nationwide Children’s Hospital, Columbus, OH) and Dr. Peter Collins (National Institutes of Health, Bethesda, MD) [20]. This virus was generated from the full-length rgRSV plasmid MP224 by replacing its first gene, which encodes the enhanced green fluorescent protein, with the wild-type *Discosoma* RFP gene from pDsRed (Clontech, Palo Alto, CA).

The rrRSV stock was propagated using HEp-2 cells grown at 37°C in 5% CO_2_ in DMEM medium supplemented with 10% fetal bovine serum and 1% penicillin/ streptomycin (Gibco). Cells at approximately 60% confluence were inoculated with a combination of 1 ml of virus stock and 4 ml of OPTI-MEM medium per flask. After a 3-hour incubation at 37°C, the inoculum was removed and replaced with 25 ml of fresh OPTI-MEM medium. The virus was harvested after 3–4 days, at which point cells appeared bright red when viewed under a fluorescent microscope. The final titer was determined with a fluorescence-focused unit titer assay. Infection of placental cells was performed at a multiplicity of infection (MOI) of 0.01 or 1.0 by adding the viral inoculum onto the cells and removing it after 3 hours, and was monitored by observing RFP production within the infected cells using an inverted fluorescence microscope (DMi8; Leica Microsystems, Buffalo Grove, IL).

To compare the proportion of placental cells supporting rrRSV replication over time, cells were infected at a MOI of 1.0 and those exhibiting RFP production under fluorescent microscopy were counted as infected every 24 hours. The number of infected cells for any given field was divided by the total number of cells for that field and multiplied by 100. A total of 8 fields was examined per each time point to calculate mean infectivity for each cell type. Isolated cytotrophoblast cells could be monitored up to 72 hours, by which point they had fused into multinucleated syncytiotrophoblast. Fibroblasts and Hofbauer cells were routinely monitored and counted up to 96 hours, except in select experiments where Hofbauer cells were maintained for up to 30 days.

### Western blot

10 μg of protein was separated in a 10% SDS-PAGE gel (BioRad, Hercules, CA) and then transferred onto a 0.45-μm PVDF membrane (Thermo Fisher, Waltham, MA). The membrane was blocked with 5% BSA in TBS-T, then probed with a rabbit polyclonal primary antibody raised against the RSV glycoprotein (G protein, GTX7038; GeneTex, Irvine, CA) diluted at 1:1,000. After washing again with TBS-T, the membrane was incubated with horseradish peroxidase-conjugated secondary antibody diluted at 1:2,000. Luminol-generated signal was detected using the MyECL imaging system (Thermo Fisher/Pierce). Anti-GAPDH (Abcam, Cambridge, UK) was used to probe for the housekeeping protein.

### Immunofluorescence

Cells were fixed with 4% paraformaldehyde for 10 min at room temperature, blocked in 5% BSA for 1 hour at room temperature, and then probed with 1:250 dilution of anti-CD45 mouse monoclonal antibody (clone HI30; BioLegend, San Diego, CA) and an identical dilution of anti-CD163 rabbit monoclonal antibody (EPR19518; Abcam, Cambridge, MA) overnight at 4°C. Donkey AlexaFluor 488 secondary antibodies were used for detection. A negative control without primary antibody was used to confirm specificity. Nuclei were stained with DAPI.

### Quantitative polymerase chain reaction (qPCR)

Cells were scraped and homogenized in RLT buffer with 1% betamercaptoethanol by vortexing and centrifugation through QiaShredder tubes at 14,000 rpm for 2 min. Total RNA was extracted using the RNeasy Mini Kit automated on QiaCube (Qiagen, Hilden, Germany). RNA from the corresponding supernatants was isolated using the QiaAmp Viral RNA kit automated on QiaCube. RNA transcripts encoding the RSV nucleocapsid (N) protein were quantified in cell homogenates and supernatants using a N gene-targeted commercial primer/probe set (PrimerDesign, Cambridge, UK), the BioRad Universal Probes One-Step qPCR kit, and 10 ng of input RNA per reaction. ß-actin was used as reference gene (IDT Assay Hs.PT.39a.22214847). N gene transcripts in the supernatants were normalized based on equal input volume. Data were obtained through the FAM channel of a CFX Connect real-time cycler (BioRad). Each sample was assayed in 3 technical replicates. Fold changes were calculated for each time point using the 24-hour value as baseline.

### Fluorescence-focused unit (FFU) assay

Hofbauer cells were infected with rrRSV at MOI of 1.0 and grown for 24 or 72 hours. After collecting the supernatants at each time point, the corresponding cells were transferred to HEp-2 medium by scraping, vortexed, passed 10 times through a 27-gauge needle, and centrifuged at 12,000xG for 10 min at 4°C to remove cellular debris. Supernatants or cell-free medium were added to HEp-2 cells at approximately 80% confluency for titration. Viral titers were multiplied by the total volume of the cell homogenates, divided by the total number of cells in the homogenate, and multiplied by 10^6^ to obtain the final titer in FFU per million Hofbauer cells.

### Hofbauer/16HBE co-infection

16HBE cells (a SV40-immortalized human bronchial epithelial cell line) were expanded in DMEM media supplemented with Pen/Strep and 10% FBS prior to use in co-culture experiments. Hofbauer cells were plated in 6-well plates and infected with rrRSV at a MOI of 0.01. After 48 hours of infection, cells were extensively washed to remove any viral progeny that might be present, and were then overlaid with 2.0x10^5^ 16HBE cells per well in DMEM/F12 media. Spread of the infection was monitored daily by observation of RFP production within the infected Hofbauer cells and naïve 16HBE cells. At 72 hours post-overlay, co-cultured cells were fixed and immunolabeled for CD45 as described above. Uninfected HBC overlaid on 16HBE cells served as negative controls.

### Cytokine analysis

Interferon alpha (IFN-alpha), transforming growth factor alpha (TGF-alpha), interleukin (IL)-4, IL-6, IL-10, IL-12, and monocyte chemoattractant protein (MCP)-1 concentrations were measured in the supernatants of Hofbauer cells infected with rrRSV at MOI of 1.0 and uninfected controls after removing cellular debris by centrifugation. All samples were analyzed at 24-hour post-infection using a Milliplex MAP Human Cytokine/Chemokine Magnetic Cytokine kit and the Magpix/Luminex platform in a multiplex format (EMD-Millipore, Burlington, MA). Tumor necrosis factor alpha (TNF-alpha) was analyzed using conventional sandwich ELISA kits (Thermo Fisher).

### Statistical analysis

Data are expressed as mean ± standard error of the mean (SEM). Fold change was calculated as the ratio of the difference between final value and the initial value over the original value. Statistical analysis of the differences in cytokines concentration was performed using the Mann-Whitney U test. GraphPad Prism version 5 for Windows (GraphPad Software, La Jolla, CA) was used for all analyses. P-value <0.05 was considered significant.

## Results

### RSV exhibits differential tropism for the three major placental cell types

**Fig 1:**
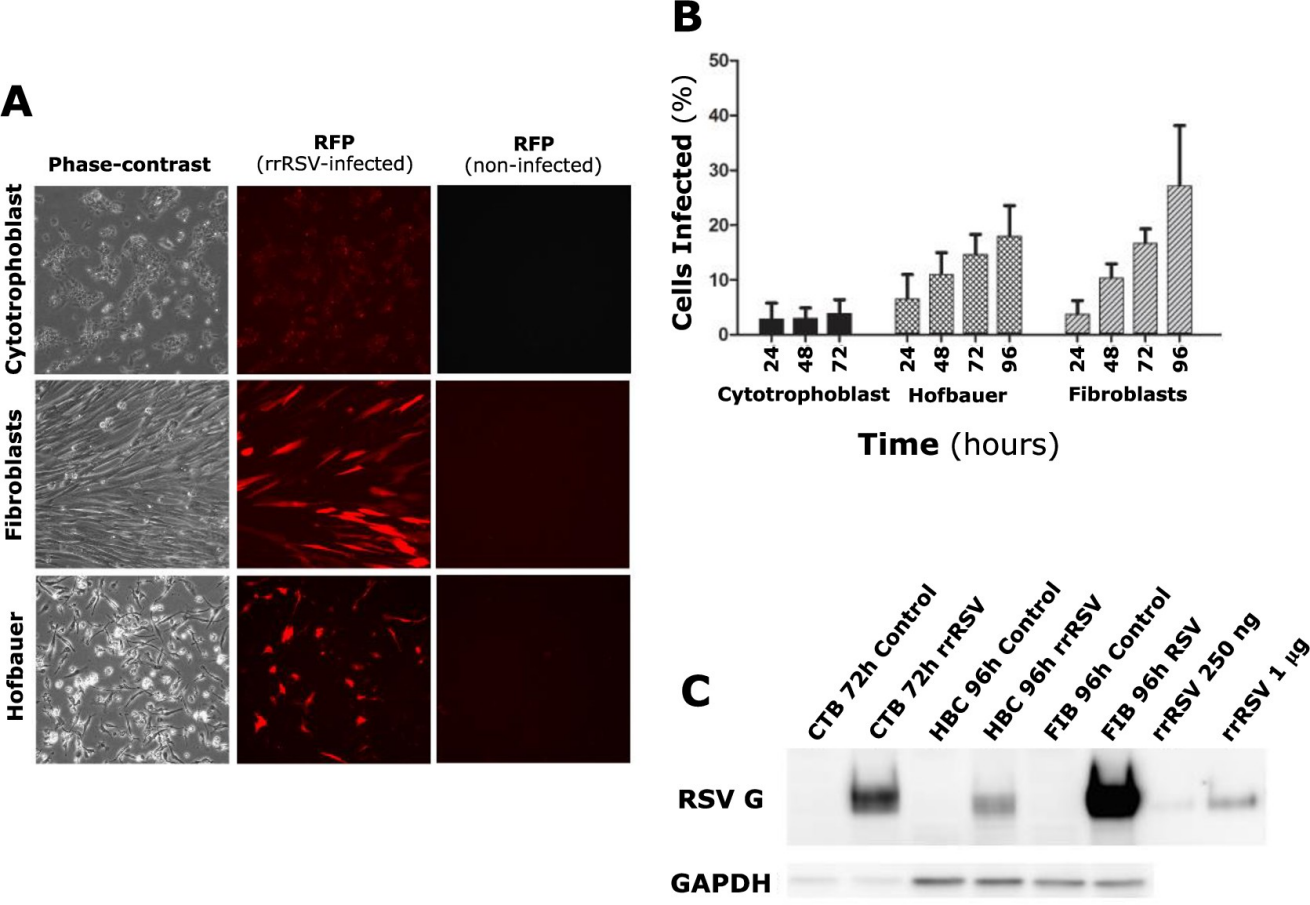
RSV infection of primary human placental cells. (A) Isolated human placental cells (left panels) after 72 hours (cytotrophoblast cells) or 96 hours (fibroblast and Hofbauer cells) infection at MOI of 1.0 with an RSV-A2 strain (rrRSV) expressing red fluorescent protein (RFP). Strong RFP expression resulting from rrRSV replication was observed in fibroblast and Hofbauer, but not in cytotrophoblast cells (center panels). Matched non-infected controls did not exhibit any fluorescence (right panels). (B) Proportion of cytotrophoblast, fibroblast, and Hofbauer cells exhibiting red fluorescence as a result of rrRSV replication. Each time-point represents the mean ± SEM of 8 different fields. (C) Expression of RSV G protein in cytotrophoblast (CTB), Hofbauer (HBC), and fibroblast (FIB) cells infected with rrRSV and processed at 72 or 96 hours for Western blot analysis. rrRSV lysate was used in two different concentrations (250 ng and 1 μg) as a positive control.

To determine whether RSV is able to infect the human placenta, primary cultures of cytotrophoblast, Hofbauer, and fibroblast cells derived from villous tissue samples were infected with rrRSV at MOI of 1.0. RFP expression was detected in both fibroblasts and Hofbauer cells as early as 24 hours post-infection and intensified through the next 48–72 hours, whereas little or no RFP was observed in cytotrophoblast cells (**Fig 1A**) S2 and S3 Tables. Also, the proportion of infected cells increased over time among fibroblasts and Hofbauer cells, but not among cytotrophoblast cells (**Fig 1B**). Placental fibroblasts appeared to be the most permissive cell type, with >20% percent infected cells by 96 hours. To assess the synthesis of viral proteins within infected cells, RSV G protein expression was analyzed by Western blot in all three cell types after 72–96 hours of infection. G protein bands were detected in each of the infected cell types, but not in uninfected controls (**Fig 1C**). Interestingly, robust G protein synthesis was observed in cytotrophoblast cells despite modest or absent RFP expression.

### Kinetics of RSV infection vary in Hofbauer cells isolated from different donors

**Fig 2:**
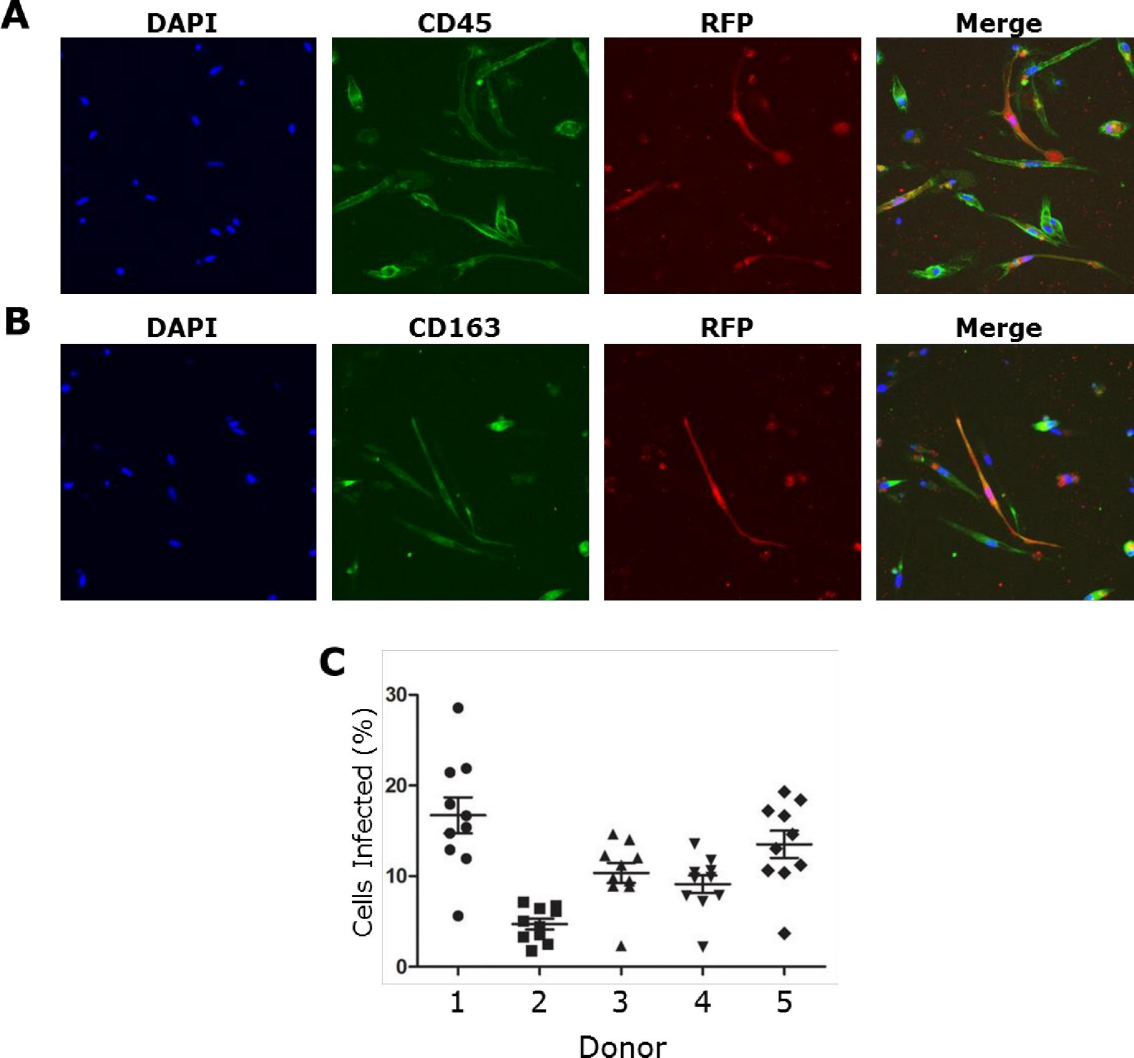
RSV infection of Hofbauer cells. Hofbauer cells were infected with rrRSV at MOI of 1 for 96 hours and then fixed with 4% paraformaldehyde. RFP expression by replicating rrRSV co-localized with (A) myeloid cell marker CD45 and (B) macrophage cell marker CD163 in infected Hofbauer cells. (C) Proportion of Hofbauer cells from 5 different donors exhibiting red fluorescence as a result of rrRSV replication. Infectivity was variable among donors and ranged from approximately 5 to 17 percent. Data are shown as mean ± SEM of 5 different donors.

As Hofbauer cells have been shown to play an important role in several placental and congenital viral infections, further experiments were performed to investigate their ability to support RSV infection. RFP expression within these cells co-localized with the myeloid cell marker CD45 and the macrophage cell marker CD163 (**Fig 2A and 2B)**. A comparison among donors revealed variability in the proportion of cells infected after 96 hours incubation, ranging from 5% to 17% (**Fig 2C**) S1 Table.

**Fig 3:**
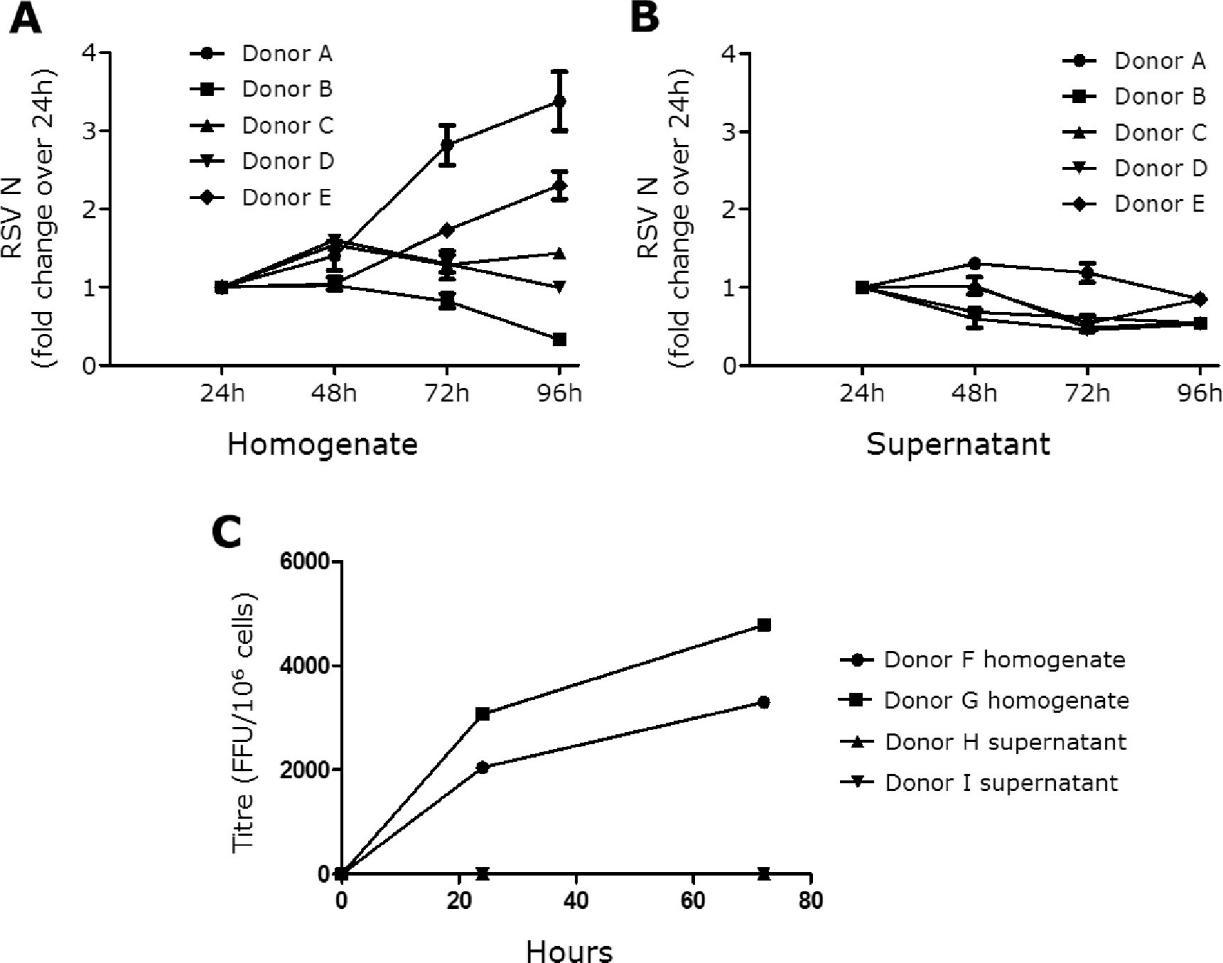
RSV infection kinetics in Hofbauer cells. (A) Expression of RSV nucleocapsid (N) RNA transcripts measured in lysates of Hofbauer cells infected with rrRSV at MOI of 1 using ß-actin as housekeeping gene. Fold changes were calculated using the 24-hour time-point as baseline to demonstrate growth kinetics between cell homogenate and supernatants. Data are shown as mean ± SEM of 5 different donors. (B) RSV N RNA transcripts in supernatants matching the cell homogenates shown in panel A and normalized based on input volume. RSV N transcription increased in cell homogenates of 4 of the 5 analyzed donors, whereas no increase was observed in the corresponding supernatants. (C) Viral titers measured at 24- and 72-hours post-infection with rrRSV at MOI of 1 using a fluorescence-focused unit (FFU) assay. Whereas viral replication within Hofbauer cells was detected at 24 hours and increased by 72 hours, no virus was found in the corresponding supernatants at either time-point.

The replication kinetics of rrRSV in Hofbauer cells was also assessed by qPCR targeting RSV N gene transcripts in cell homogenates and in the corresponding supernatants S5 and S6 Tables. Again, infected cells from 4 out of 5 donors exhibited varying degrees of elevation in N gene transcription over the course of 96 hours (**Fig 3A**). In contrast, no significant increase was detected in the corresponding supernatants (**Fig 3B**). In separate samples, viral titers were measured at 24- and 72-hours post-infection using a fluorescence-focused unit assay (**Fig 3C**) S7 Table. Whereas viral replication within cells was detected at 24 hours and increased by 72 hours, no virus was found in any of the supernatants at either time-point. It is also notable that neither syncytia formation, nor increased cell death was appreciated among infected Hofbauer cells, which remained viable for up to 30 days following infection.

### RSV may spread from Hofbauer cells through a contact-dependent mechanism

**Fig 4:**
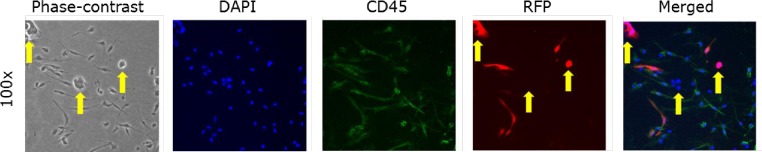
Direct transmission of RSV from Hofbauer cells to naïve 16HBE. Hofbauer cells were infected at a MOI of 0.01 and incubated for 48 hours, after which uninfected 16HBE cells were seeded for co-culture. After 72 hours of co-culture, the cells were fixed for analysis of RFP expression by fluorescence microscopy. The characteristic epithelial “colonies” formed by 16HBE cells (yellow arrows) and the CD45 staining (green) identifying Hofbauer cells allowed to recognize the two cell types. RFP expression (red) and syncytia formation was observed in 16HBE cells at 72 hours, indicating transmission of the infection from the Hofbauer cells to the epithelial cells.

Next, to determine whether RSV-infected Hofbauer cells are able to transmit virus to neighboring cells, naïve 16HBE epithelial cells were seeded onto Hofbauer cells previously infected with rrRSV at a MOI of 0.01 for 48 hours. At 72 hours post-overlay, multiple foci of strong RFP expression were observed within the 16HBE cells, with concomitant formation of multinuclear syncytia typical of RSV infection of epithelial cells (**Fig 4A and 4B**). Consistent with the experiments outlined in the previous section, Hofbauer cells did not form syncytia during co-culture with 16HBEs.

### RSV-infected Hofbauer cells secrete proinflammatory cytokines

**Fig 5:**
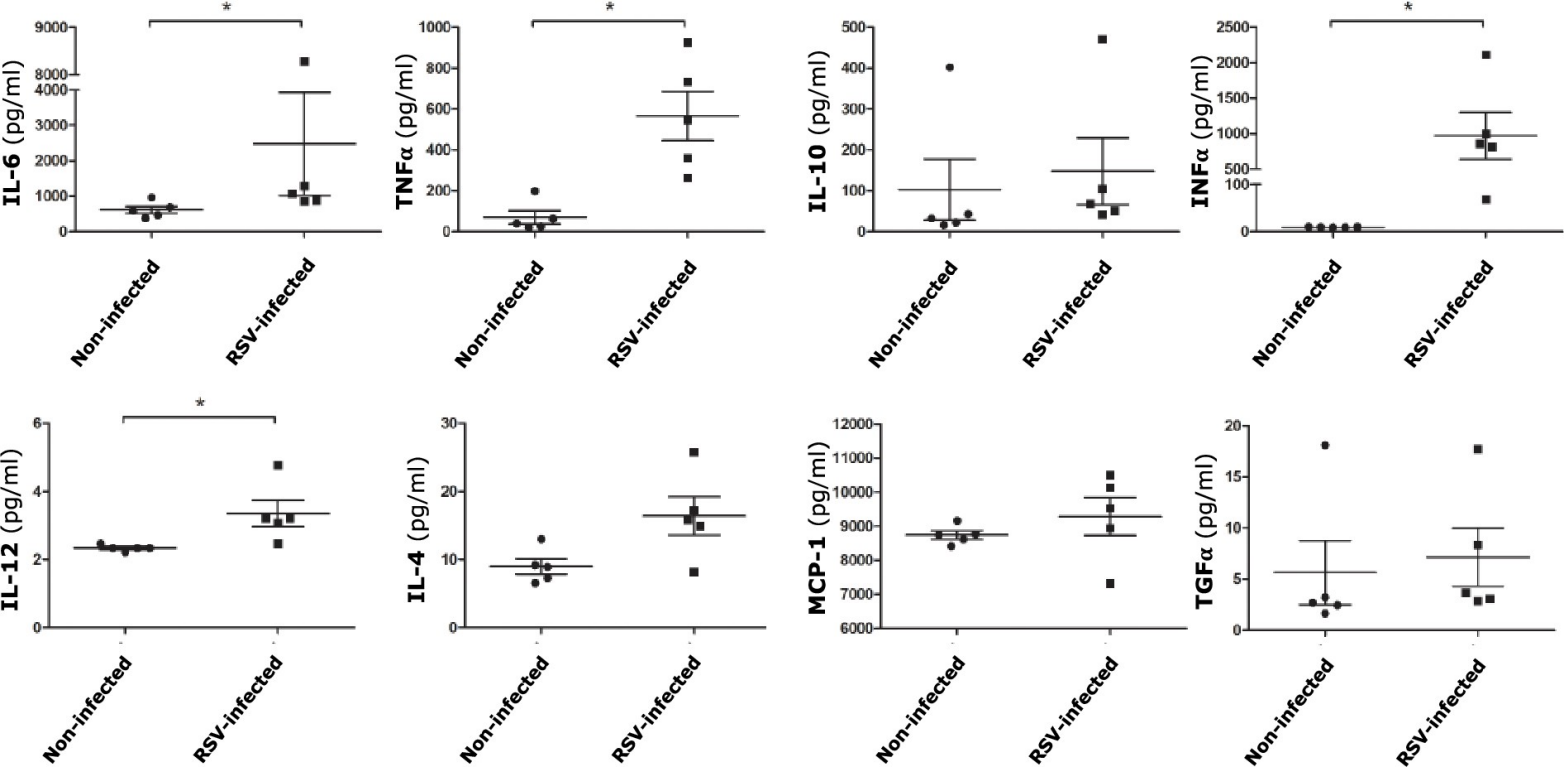
Cytokine expression of Hofbauer cells following infection with RSV. Cytokines concentration in supernatants collected at 24 hours post-infection with rrRSV at MOI of 1 were measured using the Luminex/Magpix magnetic bead platform, with the exception of TNF-alpha that was measured by conventional sandwich ELISA. The supernatants of infected Hofbauer cells exhibited significantly increased expression of the proinflammatory cytokines IL-6, TNF-alpha, IFN-gamma, and IL-12. Data are mean ± SEM; n = 5 donors; *p<0.05 vs. non-infected controls).

To characterize the inflammatory response of Hofbauer cells to RSV infection, the expression of eight cytokines was measured in uninfected controls and rrRSV-infected cells at 24 hours post-infection (**Fig 5**) S4 Table. Concentrations of TNF-alpha and IFN-gamma were markedly increased in the supernatants of infected cells (p<0.05). A less prominent, but still statistically significant increase was also measured for IL-6 and IL-12 (p<0.05). In addition, there was a trend to increased IL-4 expression that did not reach statistical significance (p = 0.056). No change was noted in the expression of IL-10, TGF-alpha, and MCP-1.

## Discussion

This study demonstrates that primary human placenta cells are effectively infected by RSV *in vitro*, even when exposed to low viral titers (MOI 0.01). To our knowledge, this is the first evidence of infection of human placenta by a respiratory virus. It also confirms and completes our previous report [21]–recently confirmed by an independent lab [22]—showing RSV infection of BeWo cells, a human choriocarcinoma cell line that retains some structural and functional characteristics of the placental trophoblast. Indeed, of the three major cell types found in the human placenta, the cytotrophoblast exhibited the lowest susceptibility to this infection, whereas fibroblasts and Hofbauer cells were significantly more permissive. Furthermore, Hofbauer cells allowed the virus to complete its replicative cycle without releasing progeny virus, and yet were able to pass the infection to neighboring epithelial cells. The translational significance of our findings is linked to the multiple lines of evidence indicating that the same cells are implicated in the transmission of several other viruses causing congenital infections.

The present study contributes an additional element to the mounting evidence that RSV can cross the fetal-placental interface, as originally postulated by our group [6]. More recently, this hypothesis has been confirmed by publication of the first documented case of congenital RSV infection caused by prenatal transmission from a mother infected during pregnancy to her newborn son [23], and also by independent research that detected RSV in 26 of 45 (58%) cord blood samples using digital droplet (dd)PCR [8]. Importantly, the new data discussed herein demonstrate susceptibility to RSV infection varies among cells derived from different donors, suggesting modulation by genetic and epigenetic traits intrinsic to the placental cells and independent from the host immune function or RSV virulence. Also, the number of cells infected *in vitro* was proportional to the size of the inoculum, implying that the risk of placental involvement *in vivo* may be contingent on severity and duration of the maternal respiratory infection and subsequent viremia.

Although infected cytotrophoblast cells do synthesize appreciable amounts of RSV G protein, they do not seem to support full replication and production of complete virions based on sparse expression of the RFP protein tag, which requires active replication of the entire viral genome to be translated. These cells serve as progenitors that undergo spontaneous fusion and subsequently differentiate into the syncytiotrophoblast, functioning as the primary interface between maternal and fetal circulation. Despite being resistant to most viral and bacterial infections [24], this placental filter can be circumvented by some pathogens using non-canonical pathways. For example, CMV crosses the syncytiotrophoblast via Fc receptor-mediated transcytosis of IgG-virion immune complexes, and subsequently replicates in other placental cell types when CMV-neutralizing antibody titers are low [10]. Interestingly, antibody protection against RSV is short-lived and incomplete in humans, even after repeated exposures [25], and studies of primary infections show the neutralizing antibody titers typically decline to pre-infection levels within a few months [26]. Also, because RSV has been located in human peripheral blood mononuclear cells, it may be shielded from immune defenses and delivered to extrapulmonary tissues within these cells [2, 3, 27], similar to the closely related Nipah virus [28]. Another mechanism by which viruses may bypass the syncytiotrophoblast involves infection of extravillous trophoblast cells—which invade through the syncytiotrophoblast layer and into decidua basalis—where they remodel spiral arteries and may expose HBC to infection [29]. This process has been hypothesized to facilitate vertical transmission of several microbial pathogens including ZIKV [30].

While placental fibroblasts are most susceptible to RSV infection, our study was primarily focused specifically on the role of Hofbauer cells, as they play a central role in many important viral infections of the human placenta. Initially thought to be protective against invading pathogens, these M2-type macrophages of fetal origin are located in the chorionic villous stroma in close association with the fetal vasculature, and are capable of harboring viruses that can be subsequently transmitted to the fetus. As stated before, Hofbauer cells are also the primary target of Zika virus [16, 18], another virus hematogenously transmitted to the fetus whose recent outbreaks have refocused our attention on the risks associated with viral infections during pregnancy. In our study however, we highlight several important differences between RSV and ZIKV with regards to their tropism and infection characteristics in Hofbauer cells.

Specifically, in the case of RSV, no infectious virus was released in the supernatants, while high viral loads were measured in cell homogenates, suggesting the virus remains trapped within the cells or on their surface. Yet, infected Hofbauer cells are still able to pass the infection to naïve epithelial cell recipients that subsequently display characteristic syncytia, suggesting viral transmission by a contact-dependent mechanism (i.e., infectious synapses). This phenomenon has been observed with measles virus [31, 32] and retroviruses such as HIV-1 [33], which are known to spread from dendritic cells to T cells by contact-dependent trans-infection. Further studies are needed to elucidate whether RSV can employ a similar mechanism to spread to certain cell types, and should focus on identifying regions of interaction between infected macrophages and naïve cells using methods such as electron microscopy. Due to the difficulties we had experienced with Hofbauer cell cultures–such as poor adherence to any surfaces other than plastic and occasional reduced viability upon isolation–there were considerable methodological limitations to our ability to characterize further their response to RSV infection.

RSV has been shown to be able to establish persistent infection in macrophage-like cell lines *in vitro* and in animal models *in vivo*, suggesting these cells may serve as viral reservoirs [34]. Our study is the first to demonstrate that human placental macrophages can support RSV infection for up to 30 days without any apparent effect on cell viability. This long-term persistence within Hofbauer cells can be explained by the extensive scientific evidence indicating that RSV upregulates multiple anti-apoptotic pathways in different host cells, including macrophages and bronchial epithelial cells [35–38]. As Hofbauer cells are also characterized by migratory behavior, it is conceivable they could serve as a “Trojan horse” mediating the transfer of RSV to other resident placental cells like the stromal fibroblasts [17], or even cross into the fetal circulation to reach the developing fetal lungs.

Placental infections often result in complications of pregnancy and negative fetal outcomes, and in this context Hofbauer cells seem to have an important role in determining fetal morbidity and mortality [39]. In particular, whereas normal pregnancies are characterized by a Th2-type cytokine bias, the release of Th1 proinflammatory cytokines from placental macrophages has been associated with numerous complications [40]. For instance, the hyperplasia of Hofbauer cells seen in CMV- and ZIKV-induced villitis is characterized by a proinflammatory cytokine pattern that contributes to placental damage [39]. Furthermore, high concentrations of TNF-alpha, IFN-gamma, and IL-6 have been measured in the amniotic fluid of mothers undergoing spontaneous miscarriage, preterm delivery, and preeclampsia [41–44]. Thus, the significant release of Th1 cytokines from RSV-infected Hofbauer cells suggests that this virus can influence pregnancy outcomes not only by direct invasion of the fetus, but also via production and release of soluble inflammatory mediators that can readily diffuse through the fetal-placental interface.

Among the limitations of this study, the complex milieu and architecture of placental villi are potentially important variables that cannot be fully recapitulated *in vitro*. Also, trans-placental RSV transmission *in vivo* might be influenced by a variety of factors, such as maternal immunity, timing and severity of the pregnant mother’s infection, integrity and overall conditions of the placenta, as well as other stochastic events. Our data, however, expand our understanding of placental susceptibility following maternal RSV viremia, and allude to potential long-term morbidities. Furthermore, our study highlights the potential of Hofbauer cells to spread RSV to the fetal circulation by virtue of their intrinsic migratory behavior and target the developing lungs, or to affect fetal development indirectly through release of proinflammatory cytokines.

Because the procedures for isolating cytotrophoblast cells have been established for several decades now, and have been widely used and reported in the literature, we did not perform biomarker labelling and relied on the observation of characteristic morphological features that separate these cells from other cell types. Another paper discussing syncytiotrophoblast culture from term placentas also omits biomarker staining of the primary cells involved in the study [45]. Additionally, other papers cited in our manuscript [11, 30] include studies involving trophoblast cells with no cytokeratin-7 or other cell biomarker immunostaining. Due to the waiting period before significant expansion of placental fibroblast cell cultures occurs after separation from DynaBeads (usually at least two weeks), it is highly unlikely that any other cell type would co-exist with the proliferating fibroblast cells, which are known to “take-over” cultures of other cell types. Also, because fibroblast cells are trypsinized and passaged at least once before being used in the experiments, the probability of contamination with other cell types is even less. Lastly, Hofbauer cell purity was assessed by both CD45, and CD163, both having been used previously for identification of this cell type.

In conclusion, our data provide evidence that human placental fibroblasts and Hofbauer cells are highly permissive to RSV infection, whereas the cytotrophoblast does not support its replication as effectively. Moreover, Hofbauer cells support long-term infection with this virus, produce and secrete proinflammatory Th1 cytokines, and are able to transfer the infection to bronchial epithelial cells through direct contact. Together with our previous studies in animal models and in humans, the results of these new experiments support the hypothesis that RSV infection can be transmitted transplacentally and modify the developmental trajectory of fetal lungs. Therefore, this research may lead to radically new prophylactic and therapeutic strategies aimed at protecting the unborn child and limit subsequent respiratory pathology in childhood.

## Supporting information

S1 Table Percent infectivity in Hofbauer cells. (XLSX)

S2 Table Percent infectivity in placental cell lysates. (XLSX)

S3 Table Percent infectivity in placental primary cells. (XLSX)

S4 Table Cytokines expression in Hofbauer cells. (XLSX)

S5 Table RSV amplification by qPCR in Hofbauer cells lysates. (XLSX)

S6 Table RSV amplification by qPCR in Hofbauer cells supernatants. (XLSX)

S7 Table RSV titer assay in Hofbauer cells. (XLSX)
